# Treatment of asthma exacerbations with the human-powered nebuliser: a randomised parallel-group clinical trial

**DOI:** 10.1038/npjpcrm.2014.16

**Published:** 2014-06-26

**Authors:** Christopher J Hallberg, M Therese Lysaught, René Antonio Najarro, Fausto Cea Gil, Clara Villatoro, Ana Celia Diaz de Uriarte, Lars E Olson

**Affiliations:** 1 Department of Biomedical Engineering, Marquette University, Milwaukee, WI, USA; 2 School of Medicine, University of Washington, Seattle, WA, USA; 3 Institute of Pastoral Studies and Neiswanger Institute of Bioethics, Stritch School of Medicine, Loyola University Chicago, Chicago, IL, USA; 4 Department of Medicine, Universidad Salvadoreña Alberto Masferrer, San Salvador, El Salvador; 5 Department of Medicine, Universidad José Matías Delgado, Antiguo Cuscatlán, El Salvador; 6 Casa de la Soladaridad Program, Santa Clara University, Santa Clara, CA, USA

## Abstract

**Background:**

Nebulisers aid the treatment of respiratory diseases, including asthma, but they require electricity and are often cost-prohibitive for low- and middle-income countries.

**Aims:**

The aim of this study was to compare a low-cost, human-powered nebuliser compressor with an electric nebuliser compressor for the treatment of mild to moderate asthma exacerbations in adults and children.

**Methods:**

This was a non-blinded, parallel-group, equivalence study, with 110 subjects between 6 and 65 years of age, conducted in the emergency department of a district hospital in Ilopango, El Salvador. Participants were assigned by random allocation to receive a 2.5-mg dose of salbutamol from the experimental human-powered nebuliser or the electric nebuliser control. All assigned participants completed treatment and were included in analysis. The study was not blinded as this was clinically unfeasible; however, data analysis was blinded.

**Results:**

The mean improvement in peak flow of the experimental and control groups was 37.5 (95% confidence interval (CI) 26.7–48.2) l/min and 38.7 (95% CI, 26.1–51.3) l/min, respectively, with a mean difference of 1.3 (95% CI, −15.1 to 17.7) l/min. The mean improvement in percent-expected peak flow for the experimental and control groups was 12.3% (95% CI, 9.1–15.5%) and 13.8% (95% CI, 9.8–17.9%), respectively, with a mean difference of 1.5% (95% CI, −3.6 to 6.6%).

**Conclusions:**

The human-powered nebuliser compressor is equivalent to a standard nebuliser compressor for the treatment of mild-to-moderate asthma. (Funded by the Opus Dean’s Fund, Marquette University College of Engineering; ClinicalTrials.gov NCT01795742.)

## Introduction

Approximately 300 million people worldwide suffer from asthma, and the incidence is growing.^1^ Annually, asthma is responsible for 15 million disability-adjusted life years lost^2^ and 180,000 deaths, most of which are preventable.^3^ Asthma in Latin America is a growing public health problem.^4,5^ In one study, asthma control in Latin America fell far short of goals set by international guidelines, with only 2.4% of patients surveyed meeting all criteria.^6^ The direct cost of treatment of uncontrolled asthma in Latin America is substantial,^7^ much of which comes from the unscheduled use of health care resources.^8^

The Global Initiative for Asthma calls for the use of rapid-acting inhaled β-agonists for treating mild-to-moderate asthma exacerbations.^9^ Salbutamol, the most common of these medications, is typically administered by metered dose inhaler or nebulisation. Global Initiative for Asthma guidelines state that the use of a metered dose inhaler with a spacer is at least equivalent to treatment with a nebuliser, provided that the patient uses the metered dose inhaler properly. Inhalers are highly portable, do not require electricity and have a shorter treatment duration than nebulisers. However, difficulty with inhaler technique has been well documented in high-income countries, even among individuals with advanced medical training, an issue not present with nebulisers where patients simply breathe continuously.^10,11^ In low- and middle-income countries, these difficulties could be further exacerbated by the dearth of trained health-care workers.

Nonetheless, nebulisers have a number of advantages. In addition to being easy to use, they are a general-purpose medical device capable of delivering a wide range of medications and vaccines, and for generating sputum samples for tuberculosis diagnosis. Jet nebulisers do require electricity or batteries in the case of hand-held types. However, given the versatility of the nebuliser, particularly in resource-limited countries, inhalers and nebulisers should be viewed as complementary, not competing, technologies.

**Figure 1:**
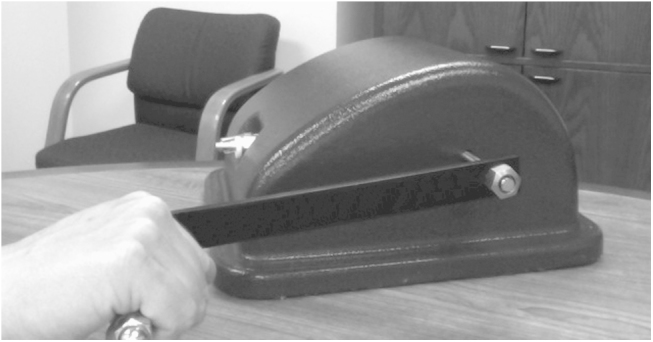
Human-powered nebuliser.

The human-powered nebuliser (HPN) is a general-purpose nebuliser compressor (Figure 1). It does not require electricity. Airflow is generated by hand cranking by a trained operator while the patient receives treatment. It was designed to be more durable and affordable than the portable ultrasonic or vibrating mesh nebulisers that carry high maintenance costs, have more complicated cleaning procedures and overheat if they are operated continuously. Laboratory experiments conducted by some of the authors of this study have confirmed that the flow rate, pressure and droplet size generated by the HPN are consistent with commercially available nebulisers (currently under review). One study has demonstrated the HPN’s effectiveness in generating sputum samples for tuberculosis diagnosis for a mobile test unit in South Africa.^12^

This paper reports the outcome of a clinical trial comparing the treatment of patients with mild-to-moderate asthma with salbutamol using the HPN and a standard electric jet nebuliser compressor (EN) at a hospital in El Salvador.

## Materials and Methods

### Setting

The study was conducted in the respiratory therapy area in the emergency department of a district hospital, Hospital Nacional San Bartolo, in Ilopango, El Salvador, during August and September 2012. All patients presenting to the respiratory area with an asthma exacerbation and a physician’s order for nebulised salbutamol were screened by the study physician for eligibility.

### Study procedure

The objective of the study was to compare the effectiveness of the HPN with an EN in the delivery of a single dose of salbutamol, for the treatment of mild-to-moderate asthma exacerbations in patients between 6 and 65 years of age, inclusive. Clinical symptoms for inclusion were based on Global Initiative for Asthma and Salvadoran Ministry of Health guidelines for mild and moderate exacerbation severity classification: (1) respiratory rate between 30 and 60 breaths per minute; (2) pulse rate between 60 and 120 beats per minute; (3) peak expiratory flow (PEF) <90% expected (best of three attempts); and (4) oxygen saturation levels >90%.

Oxygen saturation levels were measured by a pulse oximeter (CMS50F, Contec Medical System, Shanghai, China) and PEF was measured using a peak flow meter (ASSESS Peak Flow Meter, Respironics, Murrysville, PA, USA). Subjects presenting with any complicating respiratory condition and those receiving any other medication or treatment, including other asthma medications, were excluded from the study. Pregnant women and women who thought they may be pregnant were also excluded. All participants had previously been diagnosed with asthma and regularly received nebulised salbutamol for asthma exacerbations before the study. Informed consent was obtained from adult participants and from the parent of each assenting child subject.

**Figure 2:**
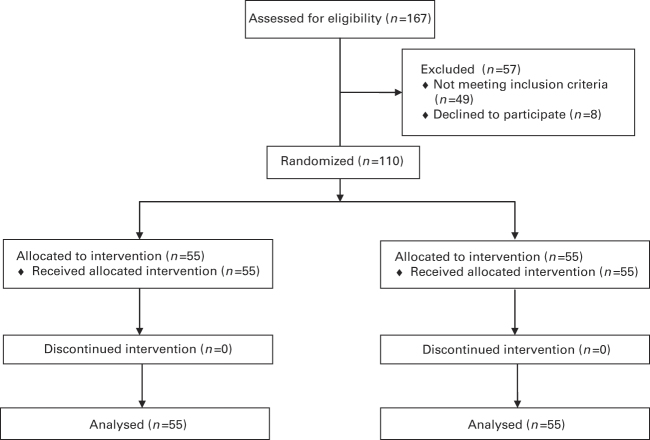
Randomisation and analysis.

This was a parallel-group trial, where each participant was randomly assigned to receive treatment with the experimental HPN compressor (Marquette University, Milwaukee, WI, USA) or the control EN compressor (DeVilbiss Pulmo-Aide 5650D, Somerset, PA, USA). Device assignment was determined by removal of a coloured tile from an opaque bag containing 55 tiles of one colour and 55 tiles of another, representing a total of 110 subjects (Figure 2). The study assistant selected the tiles and assigned each participant to the indicated group. Identical nebulisers and masks (Hudson RCI Micro Mist, Teleflex, Research Triangle Park, NC, USA) were used with both nebuliser compressors. This trial was not blinded to the participant and study care providers, as concealing the type of nebuliser compressor used was unfeasible in the clinical environment. Researchers performing data analysis were blinded to group assignment until after data analyses were performed. Each participant received a single salbutamol dose, 2.5 mg (0.5 ml of 0.5% salbutamol sulphate) in 2.5 ml of 0.9% saline in accordance with Global Initiative for Asthma and Salvadoran Ministry of Health guidelines. Both nebuliser compressors were run until completion, ~20 min. The HPN was operated by a study assistant, at a speed of ~60 revolutions per minute. The operation of the HPN does not require precise speed; an internal flow regulator ensures that the HPN produces either the prescribed airflow or none at all.

Before inclusion in the study, PEF, heart rate, respiration rate, oxygen saturation, blood pressure, temperature, height and weight of each subject was collected. During treatment, oxygen saturation and heart rate were recorded every 5 min, and the patient was under constant supervision by the study physician. On completion of the treatment, final oxygen saturation and PEF were recorded 30 min after the completion of the treatment. Increased PEF was the primary end point. Each subject was evaluated and referred to hospital staff for further treatment, if necessary.

Subjects could have been withdrawn from the study at any time, if it were determined by the study physician that the treatment was not effective for any reason. Withdrawal criteria included, but were not limited to, rapid decrease in oxygen saturation, respiratory distress and adverse reaction to salbutamol including tremors or tachycardia. Subjects could also withdraw from the study at their own discretion at any time. However, no patients from either study group were withdrawn from the study for any reason and no adverse events occurred.

### Ethical approval

The trial was conducted in compliance with the Human Subjects Institutional Review Boards of Marquette University, Milwaukee, WI, USA, and the Universidad Salvadoreño Alberto Masferrer, San Salvador, El Salvador. In addition, good clinical practices were followed.

### Statistics

The primary objective of the study was to show that the HPN was not different from EN in improving the primary outcome of improvement in PEF. The primary end point was change in PEF, reported as absolute change in PEF and change in percent-expected PEF after treatment. Change in oxygen saturation was a secondary end point. The outcome variables were compared by calculating 95% confidence intervals (CIs) for the differences between the means of the two treatment groups. Equivalence was concluded if these ranges fell entirely within ±20 l/min for absolute change in PEF or ±7% for change in percent-expected PEF. This statistical analysis has been used in previously published equivalence studies with PEF end points.^13,14^ With a two-sided alpha=0.1, a s.d. of 30 l/min for PEF improvement and a tolerance limit of 20 l/min, 52 patients were required in each group to achieve 95% power. All statistical analyses were performed in R (version 2.15.1).^15^

## Results

A total of 110 patients, 39 males and 71 females, were recruited for the study and were equally distributed between the HPN and the EN groups. Ages ranged from 6 to 65 years; all assigned participants received treatment and were included in the data analysis. For each subject, temperature and blood pressure values were all in the normal range and oxygen saturation levels were all above 90%. Other clinical measurements are presented in Table 1.

Change in PEF and oxygen saturation after treatment along with mean differences is reported in Table 2. No clinically significant differences between the HPN and EN groups were found. The change in PEF for the HPN and EN groups ranged from 40 to 170 l/min and −60 to 200 l/min, respectively. Mean pretreatment oxygen saturation values for the HPN and EN groups were 95.7% (95% CI, 95.0–96.4%) and 95.3% (95% CI, 94.7–95.9%) and post-treatment values were 97.1% (95% CI, 96.5–97.7%) and 97.1% (95% CI, 96.6–97.5%), respectively.

## Discussion

### Main findings

To our knowledge, this study is the first to compare the delivery of a therapeutic agent by a manually operated nebuliser with a commercially available jet nebuliser. This study demonstrates clinical equivalence between the HPN and the DeVilbiss Pulmo-Aide 5650D for the treatment of mild and moderate asthma.

### Interpretation of findings in relation to previously published work

The equivalence between the compressors confirms previous findings in laboratory settings. The size of the particle droplets generated by the HPN was shown to be equivalent to those generated by the EN in another study under review. In addition, the volume of liquid delivered per unit time was the same, as were mean pressure and flow from each unit. In the United States, current FDA guidance indicates that laboratory testing alone is sufficient for the approval of new electric nebuliser compressor medical devices. In the case of the HPN, however, clinical verification was considered to be an appropriate step as the human power requirement introduces an additional variable. Furthermore, this clinical study provides ‘in the field’ proof of function for Ministries of Health or non-governmental organisations interested in implementing programmes involving such a device.

### Strengths and limitations of this study

The Pulmo-Aide compressor was chosen because of its widespread use and well-characterised performance, as documented elsewhere.^16^ Certain nebuliser mouthpieces are susceptible to performance differences when not positioned vertically. To limit variability between patients, the Hudson Micro-Mist was chosen because it performs consistently at a variety of angles and has also been well studied.^17,18^ The fact that the HPN is equivalent to the Pulmo-Aide compressor suggests that the HPN performs as well as a popular and commonly used brand of nebuliser model. This equivalence at this level of performance provides evidence for Ministries of Health and global health organisations to recommend the HPN for deployment studies and further testing in regions where electricity is unreliable.

The study was limited to cases of mild and moderate asthma to minimise risk to the subjects and to ensure that patients were in a sufficiently stable condition and had ample time to decide whether or not they wished to participate. After receiving the study dose of salbutamol on either the HPN or the EN, participants were re-evaluated by the attending physician in the respiratory therapy area. Many participants received additional doses of salbutamol. The decision was made to conduct post-treatment measurements after a single dose for all participants to eliminate the complexity of adjusting for varying numbers of doses between patients. Because of these constraints, the increases in PEF and oxygen saturation levels were modest, although significantly increased.

There were a small number of subjects whose PEF or oxygenation levels did not increase. For three subjects in the EN group and two in the HPN group, PEF actually decreased slightly. The small number of non-responders in each group may have required an additional dose of salbutamol or had an undiagnosed lung pathology. Oxygen saturation improved modestly but significantly. The small level of increase is consistent with the study design of mild-to-moderate asthma symptoms. On the basis of this study and the laboratory evidence, the authors are confident that the HPN would perform equivalently to an electric nebuliser in the treatment of more severe asthma symptoms.

### Implications for future research, policy and practice

Respiratory therapy is a leading cause of clinic utilisation in El Salvador. In rural areas, patients frequently travel long distances to the nearest Ministry of Health clinic to receive nebulised treatment and often return on subsequent days for additional therapy, incurring both travel costs and lost productivity. Requiring patients to travel while having difficulty breathing could further exacerbate their symptoms, thus prolonging their recovery and increasing the number of treatments they require. With the HPN, community health workers could be trained to deliver community-based respiratory care. After the initial diagnosis by a physician in a health centre, prescribed therapies would then be delivered by the community health worker at a location near the patient’s home. Community health workers would monitor the patient and make referrals for additional treatment if necessary. This could at once reduce health centre burdens, improve the quality of care for the patient and reduce health system cost. Future research will explore the feasibility and cost of implementing such a programme in rural areas without access to electricity. In addition, the design of the HPN is open-source, and the plan for HPN deployment is to minimise cost to the users via not-for-profit mechanisms.

## Conclusions

The human-powered nebuliser compressor is equivalent to a standard nebuliser compressor for the treatment of mild-to-moderate asthma. Given the global prevalence of respiratory diseases that require a nebuliser for effective diagnosis and treatment, the implementation of a low-cost nebuliser compressor that does not require electricity could significantly reduce the social and economic burden that these diseases impose on patients and communities worldwide.

## Figures and Tables

**Table 1:** Demographic and baseline patient data

*Parameter*	*HPN (*n*=55)*	*EN (*n*=55)*
Age, years (s.d.)	27 (20)	31 (22)
Female sex, *n* (%)	36 (65)	35 (64)
Height, cm (s.d.)	147 (23)	151 (22)
Weight, kg (s.d.)	55 (26)	58 (21)
Heart rate, beats/min (s.d.)	92 (18)	91 (15)
Respiratory rate, breaths/min (s.d.)	22 (4)	22 (4)

Abbreviations: EN, electric jet nebuliser compressor; HPN, human-powered nebuliser.

**Table 2:** Outcome variables

	*HPN*	*EN*	*Mean difference*	*P-value*
Change in PEF, l/min	37.5 (26.7 to 48.2)	38.7 (26.1 to 51.3)	1.3 (−15.1 to 17.7)	0.877
Percent change in expected PEF	12.3 (9.1 to 15.5)	13.8 (9.8 to 17.9)	1.5 (−3.6 to 6.6)	0.552
Percent change in PEF	16.0 (11.1 to 20.9)	19.2 (12.3 to 26.2)	3.2 (−5.2 to 11.6)	0.451
Percent change in oxygen saturation	1.5 (0.8 to 2.1)	1.8 (1.2 to 2.4)	0.3 (−0.6 to 1.2)	0.496

Values are mean (95% CI).

Abbreviations: EN, electric jet nebuliser compressor; HPN, human-powered nebuliser; PEF, peak expiratory flow.
